# An examination of the use of standard operating procedures on family-operated farms

**DOI:** 10.3168/jdsc.2024-0587

**Published:** 2024-08-16

**Authors:** M. Beecher, T. Lawton, C. Hogan

**Affiliations:** Teagasc, Animal and Grassland Research and Innovation Centre, Moorepark, Fermoy, Co. Cork, Ireland P61 C996

## Abstract

Standard operating procedures (SOPs) can improve farm work organization by ensuring processes are standardized among the different people completing the same task. In this study, we examined the use of SOPs on family-operated farms and determined the influence of the number of people working on a farm on SOP use. A survey of 315 Irish dairy farms was completed examining the human resource and workload management practices; this study used a subset of questions from that survey. Of the 315 farms surveyed, the average herd size was 125 cows and there was 1 other person working with the farmer (range 0 to 5 people). Categorizing farms based on the number of people working on farm, 35% of the farms were owner-operators, 37% had 1 other worker, and 28% had 2 or more workers. Overall, 31.3% (n = 98) of all farms surveyed had written SOPs, whereas 68.7% (n = 217) had no written SOPs. Interest in ready-made SOPs among farmers was high (77%). Farms without SOPs found it significantly more difficult to write down work processes and lacked time to create SOPs compared with farms with SOPs. Standard operating procedures most frequently existed for milking (89%), management of fresh cows (35%), and reproduction (29%). Respondents indicated that consistency in work completion was the greatest benefit of having SOPs (39.8%; n = 39), followed by increase in efficiency (17.3%; n = 17) and health and safety (12.2; n = 12). Forty-three percent of farms with more 3 or more people had significantly more written SOPs present compared with 25% of owner-operator farms and 27% farms with 2 people. This research provides a greater understanding of the use of SOPs on family-operated dairy farms, highlighting that the presence of SOPs increases as the number of workers on farm increases.

## Highlights

• Presence of standard operating procedures on family-operated dairy farms was low.
• Standard operating procedures were most common for milking.
• As the number of farm workers increased, use of standard operating procedures increased.
• Interest in ready-made standard operating procedures was high.

## Graphical Abstract

**Figure:**
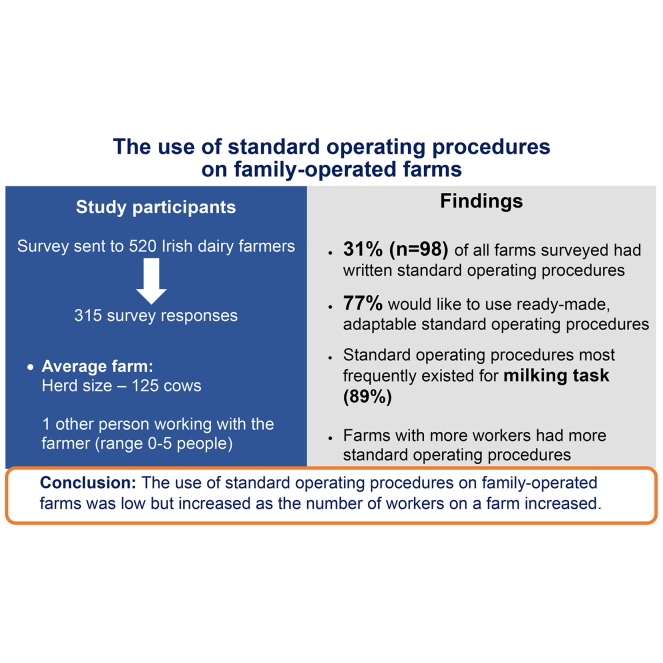
**Summary:** Three hundred fifteen Irish dairy farmers were surveyed to examine the use of standard operating procedures on family-operated farms. Respondents had an average herd size of 125 cows, and there was 1 other person working with the farmer (range 0 to 5 people). The use of standard operating procedures on family-operated farms was low but increased as the number of workers on a farm increased. Standard operating procedures most frequently existed for milking, and respondents indicated that consistency in work completion was the greatest benefit of having standard operating procedures. The high degree of interest among respondents to have ready-made, adaptable standard operating procedures suggests that there is a role for extension agents to promote the positive benefits of standard operating procedures as well as creating adaptable templates for farmers.

Family farms account for 99.7% of all Irish farms (CSO, 2016), with the labor input largely supplied by the farm household. In recent years, structural adjustments in dairy herd sizes in most Organization for Economic Co-operation and Development (**OECD**) countries have contributed to larger herds with greater requirements for labor input (Ryan, 2023). Additionally, on seasonal pastured-based dairy systems, such as those operated in Ireland, New Zealand, and parts of Australia, France, and the United Kingdom, there is a higher demand for seasonal and part-time workers during the calving and breeding period (Hogan et al., 2022). Irish dairy farms remain relatively small in scale compared with their New Zealand (average herd size 449 cows; Dairy NZ and Livestock Improvement Corporation, 2022) and Australian (average herd size 303 cows; Dairy Australia, 2022) counterparts with an average herd size of 93 cows per farm (Dillon et al., 2023). However, the increasing number of people working on Irish dairy farms, combined with ongoing workload and people management issues, have highlighted the importance of work organization and standardization of work processes to ensure that tasks are consistently completed to best practice as determined by the farmer (Hogan et al., 2023).

Work organization emphasizes the importance of managing and optimizing how work is organized and coordinated (Cordery and Parker, 2007). As farming involves many routine tasks, standard operating procedures (**SOPs**) are one aspect of work organization that can ensure work processes are standardized and consistent among the different people completing the same task (Amare, 2012). Standard operating procedures are a set of steps or written procedures to describe the most effective way that a task or activity should be completed (Hunter, 2019). The benefit of SOPs, when followed, include the minimizing of errors that may occur due to misinterpretation or miscommunication of information and reducing confusion (Amare, 2012; Barbé et al., 2016). Standard operating procedures are considered extremely important in medical and pharmacy settings but they have a value in all business settings (Amare, 2012), and are becoming increasingly required in farming situations as part of animal welfare assurance schemes (Mills et al., 2020). In addition, SOPs can be useful for training new staff (Barbé et al., 2016), especially low-skilled and migrant workers (Hesse et al., 2019).

Previous research has investigated the usefulness of SOPs related to specific tasks such as colostrum management and newborn calf care (Koralesky et al., 2021; Mills et al., 2021; Mills et al., 2020). Mills et al. (2020) conducted a qualitative study to assess the use and development of SOPs, but all farms had existing SOPs for animal care. Similarly, a German study investigated whether SOPs developed through an online microlearning course could be used to improve calf care performance (Hesse et al., 2019). All of the aforementioned studies have focused on specific aspects of SOPs or their usefulness for certain tasks; however, there is a dearth of literature regarding their use on dairy farms. Hesse et al. (2017) investigated the use of SOPs on dairy farms, but these were large farms with a median of 6 full- or part-time employees (minimum = 1; maximum = 68). There is lack of empirical evidence on the use of SOPs within farms that are largely family operated, or transitioning toward more employed labor. The objective of this study was to gain insight into the use of SOPs on Irish dairy farms and determine the influence of the number of people working on farm on the adoption of SOPs.

The survey data used in this study formed part of a larger project examining work organization and human resource management practices on Irish dairy farms. More detail regarding the survey sample selection, development, administration, and results can be found in Lawton et al. (2024) and Hogan et al. (2024).

The targeted survey population were dairy farmers from across the Republic of Ireland and aimed to be representative of herd size and location. Farmers were selected from the Irish Cattle Breeding Federation (ICBF) HerdPlus database. This database provides farmers with herd performance information and is used by ∼9,000 dairy farmers. From 6,668 farmers who granted permission for their details to be made available for research purposes, and an expected response rate of 50% to 60%, 520 farmers were selected. These farmers were selected using a stratified random sampling technique to be representative of dairy farmers in Ireland based on herd size and location. Farms were categorized into 4 herd size categories (<70 cows; 71–99 cows; 100–160 cows; and ≥160 cows) representing 25%, 23%, 26%, and 21% of the national dairy cow population, respectively (CSO, 2016) within 2 geographical regions—Border Midlands Western region, and Southern Eastern region. A sample of this size was deemed necessary to ensure a sufficient observation to cover the estimation of the coefficients for each response, and a confidence level of 95% with a CI of 5%; ensuring a robust estimation of coefficients for each response.

A questionnaire was designed based on a Dairy Australia (2017) survey that was adapted for the context of the Irish dairy industry. The survey was divided into 6 primary sections: farmer demographics, farm business and practices, farm facilities, health and safety, employee capabilities and training, and attitudes toward the industry and human resource management. The questions were primarily close-ended to obtain concise information, and structured in a format to prevent ambiguity while making it easy for the farmer to understand and complete. Care was taken to prevent bias in the design of the questions through placing focus on previously published questions in the area (e.g., Dairy Australia, 2017), and careful piloting and reviewing of survey questions. Attention was given to ensure questions were structured and phrased correctly, and to avoid leading the respondent to a given choice. Participation was voluntary and the responses were collected by mail and by telephone.

The survey was piloted with 6 farmers to determine its feasibility, comprehensiveness, and structure suitability, and appropriate alterations were made where required before distribution to the survey population. Survey packs contained the questionnaire, a cover letter, and a return-addressed envelope, and were mailed to each participating farmer between November 20 and January 3, 2019. A reminder SMS message was issued 4 wk postmailing, followed by a reminder telephone call 2 to 4 wk later. As outlined in the survey pack, farmers had the option of returning their responses via mail or completing the survey by telephone. The cover letter outlined the purpose of the research, the time frame in which surveys were to be returned, and assurance that all survey responses were confidential. Participation by farmers was completely voluntary and was not incentivized. All surveys, conducted via mail (n = 235) and telephone (n = 80), were entered into an online survey software package (http://www.surveymonkey.com). The interviewer entered the survey data to the online survey software as the farmer responded to the survey questions or after the farmer posted the survey back. Once an individual survey was completed, it was stored on the software's database with data cleaning occurring at this stage (Jones and Hidiroglou, 2013). Of the 520 farmers in the sample, the respondent population totaled 315, which was an overall response rate of 61%. Of the 315 responses, 313 had data available for analysis for the present study.

For the 6 statements outlined in Table 1 and Figure 1, respondents were asked to select from a 5-point Likert scale ranging from “strongly disagree” to “strongly agree.” For analysis purposes, responses to each statement were assigned a numerical value with 1 indicating “strongly disagree” and 5 indicating “strongly agree.” To examine the differences among these variables, the average score was calculated for a given variable.

**Table 1:** Level of agreement with statements regarding written standard operating procedures (SOPs) across farms based on whether SOPs are present or not on the farm

Item^1^	Farms with SOPs (n = 98)	Farms with no SOPs (n = 217)	Pooled SE	Mean^2^	*P*-value
On our farm different employees complete the same work processes differently.	3.7	3.7	0.70	3.7	0.72
Sometimes, I get annoyed about employees not completing tasks the way I consider right.	3.6	3.8	0.83	3.7	0.04
I have been thinking about writing down specific work processes in detail.	3.3	3.1	1.26	3.2	0.13
I find it difficult to write down work processes.	2.7	3.2	1.22	3.0	<0.001
I do not have time to create SOPs.	2.5	2.9	1.08	2.7	0.004
I would like training about how to create SOPs.	3.4	3.5	1.10	3.5	0.50

1 Mean responses were calculated based on the farmer's level of agreement with the statement, from 1 to 5, where 1 = strongly disagree and 5 = strongly agree.

2 Mean of all farms used in the analysis (n = 315).

**Figure 1:**
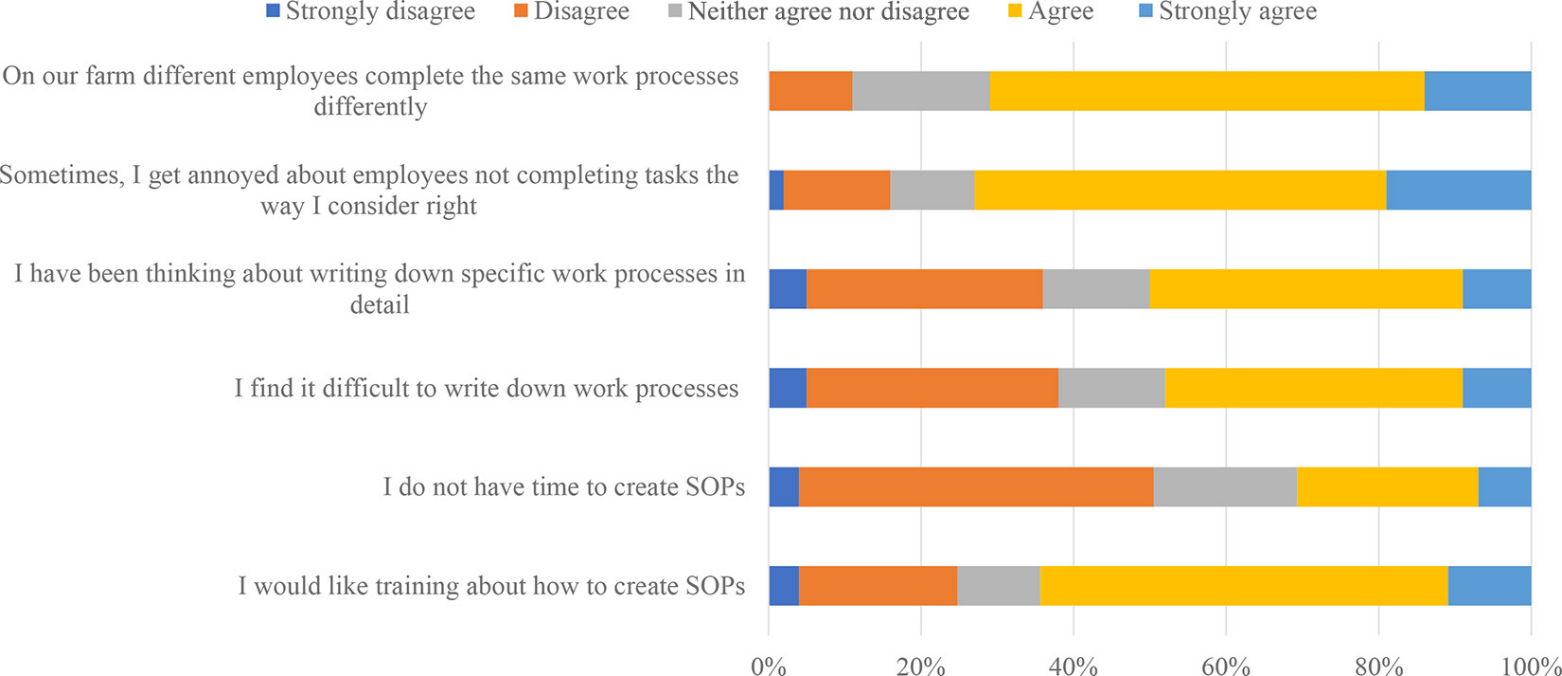
Level of agreement or disagreement with 7 statements regarding generating and implementing standard operating procedures (SOPs; n = 311).

The data were analyzed using descriptive statistics including frequencies, percentages, and means in SPSS 24 (IBM, 2016). Least squares means among farms with or without SOPs and the number of people per farm were calculated using the General Linear Model function in SPSS to examine the differences among variables. The relationships of background variables (presence of SOPs on farm and number of people working on the farm) with the type of SOPs were examined using cross-tabulation and chi-squared analysis. To examine effect of the number of people working on the farm, survey responses were categorized as 1 person (owner), owner and 1 other person, and owner and 2 or more people. Statistical differences were considered significant using a 0.05 significance level.

A detailed description of the survey population can be found in Lawton et al. (2024). Briefly, of the 315 farms surveyed, average herd size was 125 cows and there was 1 other person working with the farmer (range 0–5 people). Categorizing farms based on the number of people working on farm, 35% of the farms were owner-operators, 37% had 1 other worker, and 28% had 2 or more workers. The average farm with no SOP had a herd size of 117 cows and had 1 other person working on farm. The average farm with SOPs had a herd size of 143 cows and had 1.5 other people working on farm. The average farmer with or without SOPs were of similar age (52 yr). More farmers with SOPs (79%) had a level 5 or higher level of education compared with farmers with no SOPs (69%).

Overall, 31.3% (n = 98) of all farms surveyed had written SOPs, whereas 68.7% (n = 217) had no written SOPs. This compares with Hesse et al. (2017) who found that 54% of surveyed dairy farmers in Germany had written SOPs. However, that study indicated a much higher percentage of respondents (82%) had unwritten SOPs on farm and many did not see the importance of having written SOPs (Hesse et al., 2017), whereas in the current study, farmers were only asked about written SOPs. The relatively lower number of SOPs on Irish farms is likely to be attributed to smaller farms in size that tend to be family-run operations (Deming et al., 2019) and a reliance on short-term seasonal workers (Lawton et al., 2024). This system creates challenges working with family members, often regarding communication as the farm management styles are more likely to be informal (Kotey and Slade, 2005), more focused on day-to-day operations, and lacking consideration or discussion of the practices being conducted or what may constitute best practice for these practices. However, all farms have a potential use for, and benefit from, SOPs; for example, owner-operator businesses are managing and interacting with several people such as family members, relief and part-time workers, and agricultural contractors. Perhaps in some situations, farmers may not be aware of or see the potential benefits of SOPs (Hesse et al., 2017). This is where consultants, extension officers, and veterinarians have a role to highlight their potential, particularly as a technique that can improve communication in all farm situations as opposed to being a solution on farms where many people are employed or where communication challenges exist.

The level of agreement with statements regarding generating and implementing SOPs are presented in Figure 1. The lack of SOPs and consequently the lack of structure to a task may explain the reason why respondents had a high level of agreement (50%) with the statements, “On our farm different employees complete the same work processes differently” (71%) and “Sometimes, I get annoyed about employees not completing tasks the way I consider right” (73%). For these farmers who want a process to be completed in a certain way, implementing formal procedures including SOPs may help alleviate this challenge as they can ensure a high quality of work performance and productivity (Stup et al., 2006). This is something which respondents with SOPs may have experienced as a large proportion (60%; n = 57) of these farms selected consistency in work completed and efficiency as the main benefits of having SOPs. Overall, the adoption of SOPs on Irish dairy farms was low. Increasing the use of SOPs can have positive benefits in terms of ensuring that work processes are standardized and consistent among the different people completing the same task, making it easier to train new staff on farms. This is particularly important for dairy industries such as Ireland, where many dairy farmers are employing more people or becoming employers for the first time, and the use of SOPs has the potential to help support farmers during this transition.

Standard operating procedures most frequently existed for milking (89%), management of fresh cows (35%), and reproduction (29%). On seasonal calving pasture-based dairy farms, milking is the most time-consuming task (Deming et al., 2019; Hogan et al., 2023). Therefore, it is often a task that is completed by different people, and on Irish farms it is a common task that farmers look to external or relief labor to make more time available (FRS, 2022). Accordingly, this is likely the reason why SOPs for milking and management of fresh cows were the most common ones found on the farms surveyed. A report by the Farm Relief Services (a farmer-owned cooperative organization for the provision of skilled people to meet farm labor supply requirements) in Ireland indicated that animal husbandry and calf-rearing tasks were 2 tasks that farmers would be looking for assistance with in the future (FRS, 2022). Therefore, these tasks should be prioritized by agricultural organizations to develop resources for farmers as respondents indicated a strong desire for ready to use SOPs that could be adapted to their farm, corroborating the finding of Hesse et al. (2017). The development of adaptable resources will be important for farmers; however, it is essential that such resources give farmers control and are templates for them to tailor to suit their own needs (as opposed to the developer retaining control; Mills et al., 2020). Additionally, when engaging with someone working on the farm it is important to avoid communication problems, and a high level of management ability is required (Bewley et al., 2001; Winsten et al., 2010), which is where the use of SOPs can become an essential management tool (Barragan et al., 2016).

Consistency in work completion was selected by those farmers as the greatest benefit of having SOPs (39.8%; n = 39), followed by increase in efficiency (17.3%; n = 17) and health and safety (12.2; n = 12). Similarly, previous research identified that a benefit of SOPs included uniformity across personnel in any given task and reducing errors (Barbé et al., 2016). Farms without SOPs reported a higher level of agreement (3.8/5) with the statement “Sometimes, I get annoyed about employees not completing tasks the way I consider right” compared with farms with SOPs (3.6/5; *P* = 0.04; Table 1). Those same farms, without SOPs, found it more difficult to write down work processes and did not have time to create SOPs (*P* < 0.001; Table 1). Many farmers may lack the time to create SOPs due to a long working week relative to other industries (Hogan et al., 2023), especially when many farmers have struggled with transitioning to increased managerial and human resource management tasks following farm expansion (Hadley et al., 2002). In dairy farming, the management of time can be difficult as there are a variety of tasks, often with limited structure in the manner in which they are completed outside of the routine milking tasks (Hostiou and Dedieu, 2012). However, Hogan et al. (2023) found that improved work organization was associated with standardization as defined by having early and consistent finish times and completing fewer tasks per day. In that regard, the development of SOPs may be useful in terms of creating a structure for tasks and improved work organization. Achieving early and consistent finish times can be particularly important on family farms, as they do not offer the traditional home and work-life boundaries that exist with other jobs (Contzen and Häberli, 2021).

Forty-three percent of farms with 3 or more people had more written SOPs present compared with 25% of owner-operator farms and 27% of farms with 2 people (*P* < 0.01; Table 2). Likewise, Hesse et al. (2017) found that farms with 100 or more cows and 6 or more employees had more SOPs compared with farms with fewer than 100 cows and 3 employees. Farms with multiple people working on them have a higher requirement for training of employees, and in that regard, SOPs can be useful (Hesse et al., 2019). On larger farms, farmers spend more time on administrative tasks including managing employees (Hogan et al., 2022), which may explain why farms in our study that had more people working on the farm had more SOPs compared with farms with fewer people involved. For farms with only the farmer, observed frequencies indicated that 21% of farms (23/110) had a SOP for milking, whereas 25% of farms (29/117) with 2 people working, and 40% of farms (34/86) with 3 or more people had a milking SOP. The chi-squared test yielded significant results (χ^2^ = 9.1, *P* = 0.02, Cramér's V = 0.17). These results imply a statistically significant, albeit moderately sized, association between number of people working on farm and the presence of a milking SOP. A similar effect was observed between farms for SOPs on calf rearing (χ^2^ = 10.1, *P* = 0.01, V = 0.18) and machinery (χ^2^ = 9.5, *P* = 0.01, V = 0.18). This result is likely to be limited to the sample size used in the current study, but remains important in terms of highlighting the opportunity for increased SOP use to improve communication, particularly on farms with fewer people working on them. This is because these farms are more likely to have alternating relief staff (i.e., family members or different people completing casual work as opposed to regular part-time staff). In addition, SOPs can offer structure and routine for the farm to be operated should the main operator have to leave the farm (e.g., due to ill health or an emergency).

**Table 2:** Description of the written standard operating procedures (SOPs) present on Irish dairy farms categorized according to number of people working on the farm including the farmer

Item (n; %)	Number of people working on the farm including the farmer			Total (n)	χ^2^	*P*-value
	1 (n = 112)	2 (n = 115)	>3 (n = 86)			
Written SOPs present (yes)	27; 25^a^	32; 27^a^	37; 43^b^	96	8.7	0.01
Milking	23; 21^a^	29; 25^a^	34; 40^b^	88	9.1	0.02
Breeding	6; 6	13; 11	7; 8	26	2.4	0.52
Management of freshly calved cows	11; 10	11; 9	11; 12	33	0.8	0.78
Animal health	7; 6	7; 6	9; 11	23	1.7	0.53
Calf rearing	5; 5^a^	4; 3^a^	12; 14^b^	21	10.1	0.01
Machinery (including all-terrain vehicles)	2; 2^a^	1; 1^a^	7; 8^b^	10	9.5	0.03

a,b Different superscripts indicate significant (*P* < 0.05) differences between farms categorized according to the number of people working on them.

The large response rate to the survey allows for the generalization of results to the wider dairy farming sector in Ireland and provides a useful insight into the presence and type of SOPs on what can be considered relatively small-scale family farms. However, there are some limitations to the study; productivity data were not collected, so it is not possible to link the presence of SOPs with measures such as milk quality or animal reproduction data. In addition, data were not collected regarding the experiences of those working on farms with or without SOPs. Future work should address these limitations.

In conclusion, previous research has examined the use of SOPs on large-scale farms with several employees, and there is a lack of empirical data regarding the use of SOPs on smaller family-operated farms, which are predominant across the world. This article adds to the literature on farmers' use of SOPs and development on family-operated farms. The findings of this study suggest that overall SOP use is low on family-operated farms, but usage increases as the number of workers on a farm increases. However, all farm businesses require some SOPs as, for example, owner-operator businesses are managing and interacting with several people such as family members, relief and part-time workers, and agricultural contractors. This combined with the high degree of interest among respondents for training on how to prepare SOPs suggests that there is a role for agricultural organizations to promote the positive benefits of having SOPs and develop learning materials to support farmers to adopt them.
